# Exosomal PD-L1: New Insights Into Tumor Immune Escape Mechanisms and Therapeutic Strategies

**DOI:** 10.3389/fcell.2020.569219

**Published:** 2020-10-15

**Authors:** Kaijian Zhou, Shu Guo, Fei Li, Qiang Sun, Guoxin Liang

**Affiliations:** ^1^Department of Plastic Surgery, The First Affiliated Hospital of China Medical University, Shenyang, China; ^2^Department of Pharmaceutical Science, The First Affiliated Hospital of China Medical University, Shenyang, China; ^3^Cancer Therapy Research Institute, The First Affiliated Hospital of China Medical University, Shenyang, China

**Keywords:** exosomal PD-L1, tumor microenvironment, immune escape, antitumor immune memory, abscopal effect, biomarker, detection method, immunotherapy

## Abstract

As a classical immune checkpoint molecule, PD-L1 on the surface of tumor cells plays a pivotal role in tumor immunosuppression, primarily by inhibiting the antitumor activities of T cells by binding to its receptor PD-1. PD-1/PD-L1 inhibitors have demonstrated unprecedented promise in treating various human cancers with impressive efficacy. However, a significant portion of cancer patients remains less responsive. Therefore, a better understanding of PD-L1-mediated immune escape is imperative. PD-L1 can be expressed on the surface of tumor cells, but it is also found to exist in extracellular forms, such as on exosomes. Recent studies have revealed the importance of exosomal PD-L1 (ExoPD-L1). As an alternative to membrane-bound PD-L1, ExoPD-L1 produced by tumor cells also plays an important regulatory role in the antitumor immune response. We review the recent remarkable findings on the biological functions of ExoPD-L1, including the inhibition of lymphocyte activities, migration to PD-L1-negative tumor cells and immune cells, induction of both local and systemic immunosuppression, and promotion of tumor growth. We also discuss the potential implications of ExoPD-L1 as a predictor for disease progression and treatment response, sensitive methods for detection of circulating ExoPD-L1, and the novel therapeutic strategies combining the inhibition of exosome biogenesis with PD-L1 blockade in the clinic.

## Introduction

Programmed cell death protein-ligand 1 (PD-L1) is an immune checkpoint molecule that interacts with programmed cell death protein-1 (PD-1) to mediate immunosuppression (Ribas and Hu-Lieskovan, 2016; Alsaab et al., 2017; Sun et al., 2018; Han et al., 2020). Binding of PD-L1 to PD-1 conveys a regulatory signal to T cells and an antiapoptotic signal to tumor cells, resulting in T cells exhaustion and tumor cell survival (Dong et al., 2002; Jiang X. et al., 2019; Jiang Y. et al., 2019). It is known that, as a membrane-bound molecule, PD-L1 is expressed on the cell surface of many tumor types and the PD-1/PD-L1 pathway is considered to be a critical mechanism for immune escape and tumor progression (Quail and Joyce, 2013; Yu et al., 2016; Kakavand et al., 2017; Kythreotou et al., 2018; Cha et al., 2019). Therefore, anti-PD-1/PD-L1 inhibitors are able to induce durable tumor regression and represent a unique therapeutic strategy for patients with advanced cancers (Couzin-Frankel, 2013; Mahoney et al., 2015; Ribas et al., 2016; Yaghoubi et al., 2019). However, the effective rate of anti-PD-1/PD-L1 immunotherapy remains low (Xu-Monette et al., 2017; Chamoto et al., 2020; Hatae et al., 2020). Furthermore, patients exhibiting negative PD-L1 expression can also benefit from anti-PD-1/PD-L1 blockade (Patel and Kurzrock, 2015; Shukuya and Carbone, 2016; Shen and Zhao, 2018). Thus, neither the expression pattern of PD-L1 on the tumor cell surface alone is sufficient to account for the mechanism of tumor immune escape nor accurate for predicting the response to anti-PD-1/PD-L1 treatment, although tumor tissue PD-L1 is the only indicator authorized by the FDA (Festino et al., 2016; Wang Q. et al., 2017; Li et al., 2019c; Martinez-Morilla et al., 2020). Therefore, as an alternative to membrane-bound PD-L1, exosomal PD-L1 (ExoPD-L1) that is associated with exosomes secreted by tumor cells has been investigated recently.

Extracellular vesicles (EVs) are membrane-enveloped particles produced by almost all cell types and are classified into three subgroups: microvesicles, apoptotic bodies, and exosomes, according to their biogenesis, cellular source and biological properties (Gould and Raposo, 2013; Colombo et al., 2014; Yanez-Mo et al., 2015; Xu et al., 2016; Hessvik and Llorente, 2018; van Niel et al., 2018; Margolis and Sadovsky, 2019). Microvesicles and apoptotic bodies are large EVs (normally 100−1000 μm) and are shed directly from the plasma membrane (Gould and Raposo, 2013; van Niel et al., 2018; Margolis and Sadovsky, 2019). Exosomes are small EVs (typically 30−100 μm) generated by the endocytic pathway (Thery et al., 2006; Kowal et al., 2014; Lobb et al., 2015; Kalluri, 2016; Tkach and Thery, 2016; Tkach et al., 2018). After the fusion of endosomal multivesicular bodies (MVBs) with the plasma membrane, exosomes are secreted extracellularly (Carlton, 2010; Stoorvogel, 2015; Ha et al., 2016). Neutral sphingomyelinase type 2 (nSMase2) and Rab27a are two key enzymes in the biogenesis of exosomes (Hessvik and Llorente, 2018). They are involved in the inward budding of MVBs to form intraluminal vesicles (ILVs), the intracellular precursors of exosomes, and the transportation and fusion of the MVBs to the plasma membrane, respectively (Trajkovic et al., 2008; Ostrowski et al., 2010; Lallemand et al., 2018). Genetic and pharmacological manipulation of these enzymes offers an approach to determine the various roles of exosomes *in vitro* and *in vivo*. Increasing evidence indicates that exosomes derived from tumor cells can regulate the tumor microenvironment and promote cancer progression via their cargos, which include proteins, lipids, and nucleic acids (Anastasiadou and Slack, 2014; Lazaro-Ibanez et al., 2019; Zhang and Yu, 2019; Raimondo et al., 2020; Sahebi et al., 2020). Recent studies have demonstrated that PD-L1 also exists on the surface of exosomes generated by their parental tumor cells (Chen G. et al., 2018; Lubin et al., 2018; Ricklefs et al., 2018; Theodoraki et al., 2018b; Yang et al., 2018; Fan et al., 2019; Kim et al., 2019; Poggio et al., 2019; Cordonnier et al., 2020; Huang et al., 2020). Moreover, ExoPD-L1 can function as efficiently as PD-L1 on the tumor cell surface through direct ligation to PD-1 on the surface of lymphocytes in tumor foci (Chen G. et al., 2018; Lubin et al., 2018; Ricklefs et al., 2018; Theodoraki et al., 2018b; Yang et al., 2018; Fan et al., 2019; Kim et al., 2019; Poggio et al., 2019; Cordonnier et al., 2020; Huang et al., 2020). Surprisingly, although the cell-surface PD-L1 is low or absent, the ExoPD-L1 may be highly secreted by its parental tumor cells that are resistant to anti-PD-L1 therapy (Poggio et al., 2019). Overall, ExoPD-L1 plays a pivotal role in immunosuppression and tumor progression.

In this review, we summarize the various functions of ExoPD-L1 secreted by tumor cells, focusing on the recent findings regarding their expression heterogeneity, the impact on local and systemic immune response, and tumor growth. Moreover, we also discuss the clinical implications of circulating ExoPD-L1 as a non-invasive biomarker to predict tumor progression and immunotherapeutic response, and as a novel target to develop more effective antitumor strategies.

## The Expression Pattern of Tumor ExoPD-L1

It is well known that the PD-L1 protein is abundantly expressed on the cell surface of various cancers (Cimino-Mathews et al., 2016; Nduom et al., 2016; Brody et al., 2017; Sunshine et al., 2017). Recent studies have shown that tumor cells can secrete PD-L1 in EVs, particularly in exosomes, which are generally present in the pellet obtained by ultracentrifugation (Chen G. et al., 2018; Yang et al., 2018; Kim et al., 2019; Poggio et al., 2019). Colocalization of PD-L1 and exosomal marker CD63 in MVBs is observed in human breast cancer tissues by immunohistochemical staining (Pols and Klumperman, 2009; Khushman et al., 2017; Farooqi et al., 2018; Yang et al., 2018). Furthermore, human ExoPD-L1 was found in the circulation of nude mice bearing human melanoma xenografts (Chen G. et al., 2018). Thereby, both human and murine tumor cells can secrete ExoPD-L1 both *in vitro* and *in vivo*.

The expression of ExoPD-L1 is highly heterogeneous in tumor cells. The variability in the levels of ExoPD-L1 is quite significant between different tumor types and even between different cell lines of the same type (Table 1). In addition, it appears that ExoPD-L1 levels are consistent with the levels of PD-L1 expressed in their parental tumor cells (Chen G. et al., 2018; Ricklefs et al., 2018; Fan et al., 2019; Kim et al., 2019). However, an exception is observed in prostate cancer. These tumor cells produce high levels of PD-L1-containing exosomes, but are devoid of PD-L1 on the tumor cell surface, despite expressing constitutively high levels of PD-L1 mRNA (Poggio et al., 2019). Considering the discordance between exosomal and cell-surface PD-L1 expression, the expression pattern of ExoPD-L1 from tumor cells, especially that of low or undetectable cell-surface PD-L1, should not be neglected. In addition, interferon-γ, a typical inflammatory cytokine, upregulates ExoPD-L1 production by melanoma, breast cancer, prostate cancer, glioblastoma, and non-small cell lung carcinoma (NSCLC) (Chen G. et al., 2018; Monypenny et al., 2018; Ricklefs et al., 2018; Poggio et al., 2019). However, the mechanism regulating ExoPD-1 release is not fully understood. Thus, endeavors to further explore the molecular mechanisms regulating ExoPD-L1 expression are warranted.

**TABLE 1:** Expression of ExoPD-L1 secreted by human and mouse tumor cell lines.

Tumor	High	Low	Negative	References
Breast cancer	MDA-MB-231#			Chen G. et al., 2018; Yang et al., 2018
	BT549, 4T1*	MCF-7		Yang et al., 2018
			HCC1954#, 67NR*#, SKBR&	Monypenny et al., 2018
Colon cancer	RKO			Yang et al., 2018
	MC38*			Poggio et al., 2019
Gastric cancer	MKN74	SGC7901, BGC823, NCI-N87, NUGC4, MKN45	KATOIII, AGS, MGC803	Fan et al., 2019
Glioblastoma	G34, G35, CT2A*	G44#, G157#		Ricklefs et al., 2018
Melanoma	WM9#, WM164#, UACC-903	WM1552C, WM35, WM793, WM902B	MEL624	Chen G. et al., 2018
		SK-MEL-28		Poggio et al., 2019
	B16-F10*			Chen G. et al., 2018; Cordonnier et al., 2020
	SK-MEL-2			Cordonnier et al., 2020
	A375			Chen G. et al., 2018; Huang et al., 2020
			A375	Yang et al., 2018
NSCLC	H1299#, H358#, H1264#			Chen G. et al., 2018
	H460, H1975	A549	LLC-1*	Kim et al., 2019
	HCC827			Yang et al., 2018
	A549			Cordonnier et al., 2020
Prostate cancer	PC3#, TRAMP-C2*#		LNCaP	Poggio et al., 2019

*, murine; #, IFN-γ inducible PD-L1 expression; &, IFN-γ non-inducible PD-L1 expression.

## The Immunosuppressive Effects of ExoPD-L1

The modulatory effect of tumor cell PD-L1 occurs through binding to PD-1. PD-L1 is a typical transmembrane protein (Dong et al., 1999, 2002). Recent studies reveal that ExoPD-L1 displays the same extracellular domain topology as its cell-surface counterpart (Chen G. et al., 2018). Therefore, ExoPD-L1 may exert a similar function as tumor cell-surface PD-L1 by engaging with PD-1.

### The Interaction Between ExoPD-L1 and PD-1 on T Cells

#### ExoPD-L1 Directly Binds to PD-1 on T Cells

*In vitro* binding assays showed that PD-L1 on melanoma-derived exosomes is able to ligate to soluble PD-1 molecules in a concentration-dependent manner (Chen G. et al., 2018; Ricklefs et al., 2018; Yang et al., 2018). Consistently, both PD-L1 and PD-1 blocking antibodies can disrupt the ligation in a dose-dependent manner (Chen G. et al., 2018; Ricklefs et al., 2018). The physical combination of melanoma exosomes and T cells was confirmed by using confocal microscopy, flow cytometry and enzyme linked immunosorbent assay (ELISA) (Chen G. et al., 2018; Ricklefs et al., 2018). The binding of melanoma-derived exosomes to CD8^+^ T cells is increased when the levels of either PD-1 on CD8^+^ T cells or ExoPD-L1 are upregulated (Chen G. et al., 2018). Studies on glioblastoma-derived exosomes also show that ExoPD-L1 binds to CD4^+^ and CD8^+^ T cells (Ricklefs et al., 2018). Furthermore, the *in vivo* colocalization of ExoPD-L1 to tumor-infiltrating lymphocytes (TILs) in mouse glioblastoma tissues was visualized (Ricklefs et al., 2018). Thus, ExoPD-L1 can ligate to PD-1 on T cells, which is an alternative pathway to membrane-bound PD-L1 interacting with its receptor PD-1.

#### ExoPD-L1 Interacts With PD-1 on T Cells After Migration to PD-L1-Negative Tumor Cells

It has been demonstrated that exosomes can transfer specific proteins, nucleic acids, and lipids from donor cells to recipient cells, thereby influencing the phenotype of the recipient cells (Milane et al., 2015; Ruivo et al., 2017; Wan et al., 2018; Lazaro-Ibanez et al., 2019). Recent studies found that tumor-derived exosomes can transport PD-L1 from PD-L1-positive tumor cells to PD-L1-negative tumor cells (Yang et al., 2018). After a 24 h incubation with ExoPD-L1 derived from breast cancer cells with constitutive PD-L1 expression, high levels of PD-L1 were detected in breast cancer cells with PD-L1 knockdown or low PD-L1 expression (Yang et al., 2018). Notably, the ExoPD-L1 migration to PD-L1-negative tumor cells was detectable in tumor masses of mice 5 days after coinjection of ExoPD-L1 (Yang et al., 2018). Furthermore, ExoPD-L1 can be transported to immune cells, including human macrophages and dendritic cells *in vitro* and murine tumor-infiltrated macrophages *in vivo* (Yang et al., 2018). More importantly, results obtained from flow cytometric analysis demonstrated that the ExoPD-L1, which settled on the surface of the PD-L1-negative tumor cells, is capable of binding to the PD-1 Fc fragment (Yang et al., 2018). Thus, the ExoPD-L1 that migrates to the surface of recipient cells from PD-L1-positive tumor cells still maintains its ability to bind to PD-1 on T cells (Yang et al., 2018).

Notably, CD80 is also a binding partner of PD-L1 and competes with PD-1 for engaging PD-L1 (Butte et al., 2008; Park et al., 2010; Chen and Flies, 2013). The interaction of PD-L1 on tumor cells and CD80 on T cells suppresses T cell activation and survival, suggesting that dual blocking PD-1 and CD80 interaction with PD-L1 might be more favorable for improving the immunotherapy efficacy compared with single PD-1 blockade (Butte et al., 2007; Rollins and Gibbons Johnson, 2017). In addition, PD-L1 can interact in *cis* with CD80 on the same cell (Chaudhri et al., 2018). The *cis*-heterodimer of PD-L1 and CD80 on antigen presenting cells is able to restrict PD-1 function and is the requirement for triggering T cell responses (Sugiura et al., 2019; Zhao Y. et al., 2019; Mayoux et al., 2020). Therefore, it is necessary to dissect the functions contributed by the crosstalk between PD-L1/PD-1 and PD-L1/CD80 pathways in tumor microenvironment to explore new opportunities for tumor treatment.

The PD-1/PD-L1 signaling pathway in activated T cells has been reviewed in recent literatures (Ai et al., 2020; Bastaki et al., 2020; Wu et al., 2020). After PD-1 binds to PD-L1, the cytoplasmic tail of PD-1 is phosphorylated and recruits Src homology phosphatase 1 (SHP-1) and SHP-2 (Chemnitz et al., 2004; Yokosuka et al., 2012; Chinai et al., 2015). SHP-2 dephosphorylates and inhibits T cell receptor (TCR) and downstream signaling, such as zeta-chain-associated protein kinase 70 (ZAP70), phosphoinositide 3-kinase (PI3K), protein kinase B (PKB/AKT), mammalian target of rapamycin (mTOR), rat sarcoma (RAS), mitogen-activated protein kinase (MAPK/MEK), and extracellular regulated protein kinase (ERK) (Sheppard et al., 2004; Parry et al., 2005; Riley, 2009; Patsoukis et al., 2012; Hui et al., 2017). Additionally, recent studies reported that CD28, rather than the TCR, is the primary target of SHP-2 (Hui et al., 2017; Kamphorst et al., 2017), suggesting that PD-1 may target both TCR and CD28 to exert regulatory function. It has been shown that ExoPD-L1 released by breast cancer cells significantly suppresses ERK phosphorylation and nuclear factor kappa-B activation in CD3/CD28-activated T cells (Yang et al., 2018). However, whether other molecules participate in the ExoPD-L1 signaling, especially in the context of tumorigenesis, remains unclear and needs to be further investigated.

### The *in vitro* Immunosuppressive Effects of ExoPD-L1

It has been reported that tumor-derived exosomes contribute to CD8^+^ T cell dysfunction, although the mechanism is not fully understood (Ludwig et al., 2017; Maybruck et al., 2017; Huang et al., 2018; Wang T. et al., 2019). Recent studies found that ExoPD-L1 secreted by tumor cells can efficiently induce T cell dysfunction via interacting with its surface PD-1 (Table 2).

**TABLE 2:** The inhibitory effects of tumor cell-derived ExoPD-L1 on T cells *in vitro*.

Cell source of ExoPD-L1	Target cell	Effect	Indicator	References
Human MDA-MB-231 breast cancer cells	PBMCs	Suppression of T cell activation	IL-2 ↓	Yang et al., 2018
Human RKO colon cancer cells	PBMCs		IL-2 ↓	Yang et al., 2018
Human HCC827 NSCLC cells	PBMCs		IL-2 ↓	Yang et al., 2018
Human PC3 prostate cancer cells	Jurkat T cells		IL-2 ↓	Poggio et al., 2019
Human NSCLC primary cells	CD8^+^ and Jurkat T cells		IL-2 ↓, IFN-γ↓	Kim et al., 2019
Human WM9 melanoma cells	CD8^+^ T cells		IL-2 ↓, IFN-γ↓, TNF-α↓	Chen G. et al., 2018
Human SK-MEL-2 melanoma cells	PBMCs		IFN-γ↓, PD-1 ↓	Cordonnier et al., 2020
Human MKN74 gastric cancer cells	PBMCs		CD69 ↓, PD-1 ↓	Fan et al., 2019
Human glioblastoma primary cells and murine CT2A cells	CD8^+^ and CD4^+^ T cells		CD69 ↓, CD25 ↓, PD-1 ↓	Ricklefs et al., 2018
Human glioblastoma primary cells and murine CT2A cells	CD8^+^ and CD4^+^ T cells	Inhibition of T cell proliferation	CFSE ↓	Ricklefs et al., 2018
Human WM9 melanoma and murine B16-F10 cells	CD8^+^ T cells		CFSE ↓, Ki67 ↓	Chen G. et al., 2018
Human NSCLC primary cells and H1264 cells	CD8^+^ T cells		CFSE ↓, Ki67 ↓	Chen G. et al., 2018
Human SK-MEL-2 melanoma cells	PBMCs		Ki67 ↓	Cordonnier et al., 2020
Human H1264 NSCLC cells	CD8^+^ T cells	Suppression of T cell Cytotoxicity	GzmB ↓	Chen G. et al., 2018
Human MDA-MB-231 breast cancer cells	PBMCs		Tumor-cell killing ability ↓	Yang et al., 2018
Human WM9 melanoma and murine B16-F10 cells	CD8^+^ T cells		Tumor-cell killing ability ↓, GzmB↓	Chen G. et al., 2018
Human NSCLC cells	CD8^+^ T cells	Inhibition of T cell survival	Apoptosis ↑	Kim et al., 2019
Human HNSCC primary cells	CD8^+^ T cells		Apoptosis ↑	Theodoraki et al., 2018a

*↑, increase; ↓, decrease.*

Protein-1 is mainly expressed on activated T cells and it is a central inhibitory receptor that regulates CD8^+^ T cell dysfunction in tumors (Ahn et al., 2018; Miller et al., 2019). Recent studies found that the activation-signaling pathway in T cells is inhibited in a dose-dependent manner from exposure to ExoPD-L1 derived from breast cancer cells (Yang et al., 2018). Furthermore, the production of IFN-γ, IL-2, and TNF-α by CD8^+^ T cells was decreased in the presence of ExoPD-L1 derived from melanoma, breast cancer, NSCLC, and prostate cancer (Chen G. et al., 2018; Yang et al., 2018; Kim et al., 2019; Poggio et al., 2019; Cordonnier et al., 2020). In addition, ExoPD-L1 shuts down the expression of activation markers on CD4^+^ and CD8^+^ T cells, including CD69, CD25, and PD-1 (Ricklefs et al., 2018; Fan et al., 2019; Cordonnier et al., 2020). Pretreatment with anti-PD-1 antibodies or PD-L1 knockdown constructs significantly diminished the suppression of T cell activation mediated by ExoPD-L1 (Chen G. et al., 2018; Ricklefs et al., 2018; Theodoraki et al., 2018b; Yang et al., 2018; Fan et al., 2019; Kim et al., 2019). Importantly, exosomes from plasma of patients with NSCLC and headneck squamous cell carcinoma (HNSCC) display modulatory effects on T cells (Theodoraki et al., 2018a; Kim et al., 2019). Remarkably, ExoPD-L1 from tumors is not only as efficient as cellular PD-L1, but also stronger than soluble PD-L1 in suppressing T cell activation because of the high stability of ExoPD-L1 and MHC-I expression (Fan et al., 2019; Cordonnier et al., 2020).

Recent studies showed that exosomes derived from human melanoma and NSCLC significantly reduced the Ki-67 expression of T cells and CD8^+^ T cells, which is restored in the presence anti-PD-1 blocking antibodies (Chen G. et al., 2018; Cordonnier et al., 2020). Additionally, exosomes from human glioblastoma culture block both CD8^+^ and CD4^+^ T cell proliferation (Ricklefs et al., 2018). Notably, ExoPD-L1 from NSCLC and HNSCC cells induced apoptosis in CD8^+^ T cells and the amount of CD8^+^ T cells decreased in a dose-dependent manner (Theodoraki et al., 2018a; Kim et al., 2019). Overall, tumor-derived ExoPD-L1 is able to suppress the proliferation and survival of T cells, which contributes to T cell dysfunction (Li et al., 2019b; Xia et al., 2019).

The cytotoxicity of functional effector T cells is responsible for killing cancer cells and eradicating tumors. Exosomes secreted from melanoma and NSCLC cells that express endogenous PD-L1 inhibit the expression of granzyme B (GzmB) from human peripheral and mouse splenic CD8^+^ T cells activated by TCR stimulation (Chen G. et al., 2018). Pretreatment with exosomes from tumors, such as melanoma and breast cancer, significantly inhibited the cytotoxic T cell-mediated tumor killing, which could be counteracted by anti-PD-L1 antibodies (Chen G. et al., 2018; Yang et al., 2018; Kim et al., 2019). Thus, tumor-derived ExoPD-L1 is capable of inhibiting T cell function by modulating the proliferative capacity and effector function of T cells (Table 2).

Together, ExoPD-L1 secreted from tumor cells is able to mediate immunosuppression *in vitro*. The expression of inhibitory receptors is also a characteristic of T cell dysfunction, except for low proliferation and loss of effector function (Xia et al., 2019). Therefore, in addition to PD-1, the impact of ExoPD-L1 on other T cell inhibitory receptors should be investigated, including T cell immunoglobulin domain and mucin domain-3 (TIM-3), cytotoxic T lymphocyte antigen 4 (CTLA-4), lymphocyte activation gene 3 (LAG-3), T cell immunoreceptor with Ig and ITIM domains (TIGIT) (Anderson et al., 2016; Andrews et al., 2019; Wolf et al., 2019).

### The Immunoinhibitory Effects of ExoPD-L1 in Mouse Tumor Models

**FIGURE 1:**
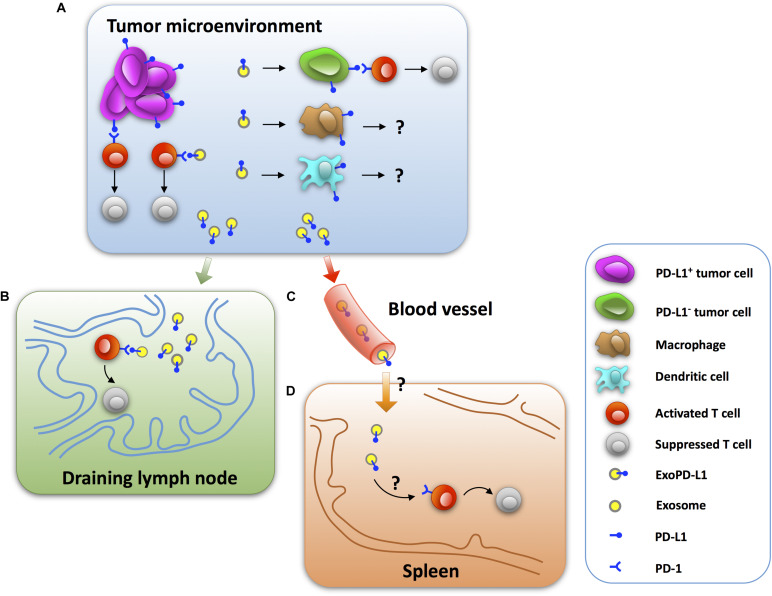
The mechanisms by which tumor ExoPD-L1 induce immunosuppression. **(A)** ExoPD-L1 originating from tumor cells induces T cell dysfunction in the tumor microenvironment by directly ligating to PD-1 on T cells as well as stationing on PD-L1-negative tumor cells, while its immunoregulatory effect remains intact. ExoPD-L1 also migrates to macrophages and dendritic cells, but the potential effects remain unknown. **(B)** ExoPD-L1 is able to leave tumor foci and enter the draining lymph node to mediate T cell suppression. **(C)** ExoPD-L1 can enter and circulate in the blood. **(D)** ExoPD-L1 inhibits the immune response in spleen and decreases spleen size.

Insight into the immunosuppressive effects of ExoPD-L1 *in vivo* is beneficial to understanding the mechanisms of tumor immune escape. Recent studies have shown that ExoPD-L1 released by tumor cells induces immunosuppressive activities at tumor sites in a paracrine-dependent manner (Chen G. et al., 2018; Yang et al., 2018; Kim et al., 2019). Additionally, exosomes can enter blood and circulate systemically, which may help ExoPD-L1 to function at distant sites in a manner similar to endocrine molecules (Figure 1; Seo et al., 2018; Wortzel et al., 2019).

#### Decreased Frequencies and Activities of TILs

A significant reduction in the number of CD8^+^ TILs was observed in melanoma tumors of C57BL/6 mice after 24 days of an injection with PD-L1-containing exosomes (Chen G. et al., 2018). Moreover, the frequency of Ki67^+^PD-1^+^CD8^+^ T cells in the tumor microenvironment decreased significantly, which was reversed by anti-PD-L1 antibodies (Chen G. et al., 2018). There was also a significant loss of CD8^+^ TILs in the tumor area of NSCLC after an intravenous injection of PD-L1-containing exosomes in mice after 14 days (Kim et al., 2019). Furthermore, PD-L1-containing exosomes reduce cytotoxic T cell activity, as assessed by GzmB expression, in the tumor microenvironment (Yang et al., 2018). Collectively, these results suggest that ExoPD-L1 plays a key role in induction of immune escape in tumor microenvironment (Figure 1).

#### Suppression of T Cell Function in the Draining Lymph Node

It is clear that exosomes are important communicators between tumors and immune cells and they exert modulatory effects on the systemic immune response (Hood et al., 2011; Groot Kormelink et al., 2018; Czystowska-Kuzmicz et al., 2019; Sheehan and D’Souza-Schorey, 2019). Recent studies reported that PD-L1-deletion significantly increases the number, proliferation (Ki67), and effector function (GzmB) of CD8^+^ T cells, while decreasing the exhaustion (TIM-3) of CD8^+^ T cells in the draining lymph node of mice injected with TRAMP-C2 prostate cancer cells (Poggio et al., 2019). Meanwhile, similar to PD-L1-deletion, Rab27a-deletion, which inhibits the biogenesis of exosomes, has a promotional effect on the frequency and activity of CD8^+^ T cells (Poggio et al., 2019). More importantly, the administration of exogenous exosomes derived from wild-type (Newton et al., 2016) prostate cancer cells leads to immunosuppression in the draining lymph node, as evidenced by a reduced number and effector function, and increased exhaustion of CD8^+^ T cells of mice injected with Rab27a-deleted prostate cancer cells (Poggio et al., 2019). Moreover, the administration of WT melanoma cell-derived exosomes significantly reduced the proportion of Ki67^+^PD-1^+^CD8^+^ T cells in the draining lymph node of mice injected with the PD-L1-deleted melanoma cells, which was counteracted by anti-PD-L1 antibodies (Chen G. et al., 2018). Thus, these findings indicate that tumor-derived ExoPD-L1 is capable of patrolling the draining lymph node and regulating T cell activation (Figure 1).

#### Reduction of Spleen Size and T Cell Proliferation in Spleen

In addition to inducing immune suppression in the draining lymph node, ExoPD-L1 can inhibit the immune response in the spleen. Recent studies reported that PD-L1-deletion significantly increases the spleen size of mice injected with TRAMP-C2 prostate cancer cells (Poggio et al., 2019). Meanwhile, Rab27a-deletion also increases the spleen size of mice injected with prostate cancer cells. Notably, intravenous injection of exogenous exosomes derived from WT prostate cancer cells resulted in decreased spleen size of mice injected with Rab27a-deleted prostate cancer cells to nearly 50% (Poggio et al., 2019). In addition, administration of WT melanoma B16-F10 cell-derived exosomes significantly reduced the proportion of Ki67^+^PD-1^+^CD8^+^ T cells in murine spleen injected with the PD-L1-deleted melanoma cells. This effect could be counteracted by anti-PD-L1 antibody treatment (Chen G. et al., 2018). Thus, ExoPD-L1 secreted by tumor cells can enter into the circulation to inhibit the antitumor immunity systemically (Figure 1).

#### Promotion of Tumor Growth Across Different Tumor Types in an Immune-Dependent Manner

It has been shown that ExoPD-L1 derived from tumor cells promotes tumor growth *in vivo*, including cancers of the breast and prostate, colorectal cancer, melanoma, and NSCLC (Chen G. et al., 2018; Yang et al., 2018; Kim et al., 2019; Poggio et al., 2019). PD-L1 knockout leads to substantial tumor regression or even failure to grow, however, this was reversed by local or intravenous injection of exosomes derived from WT tumor cells (Chen G. et al., 2018; Yang et al., 2018; Poggio et al., 2019). Different from PD-L1 deletion, which downregulates the transcripts of PD-L1 mRNA, genetic deletion of Rab27a or nSMase2 reduces the production of ExoPD-L1 via inhibition of exosome biogenesis. *In vitro* studies showed that blockade of Rab27a or nSMase2 does not change cell proliferation in prostate cancer, colorectal cancer, or breast cancer, indicating that blockade of exosome biogenesis itself does not cause the suppression of tumor growth (Yang et al., 2018; Poggio et al., 2019). However, deletion of either Rab27a or nSMase2 significantly inhibits tumor growth in mice, including that of breast, prostate, and colorectal cancers (Yang et al., 2018; Poggio et al., 2019). These findings reveal that the inhibition of exosome biogenesis or PD-L1 deletion results in a similar suppressive effect on tumor growth. In addition, prostate and breast cancer cells experiencing a blockade of Rab27a, nSMase2 or PD-L1 failed to grow in WT mice, but grow rapidly in immunodeficient mice (Yang et al., 2018; Poggio et al., 2019). Collectively, ExoPD-L1 promotes tumor growth that is dependent on the inhibition of the antitumor immune response.

### ExoPD-L1 Contributes to the Immune Suppression in Tumor Patients

It has been observed that circulating ExoPD-L1 level is positively associated with its ability to suppress the activation of CD8^+^ T cells in HNSCC patients (Theodoraki et al., 2018b). Additionally, in metastatic gastric tumor patients, the levels of plasma ExoPD-L1 are negatively associated with CD4^+^ and CD8^+^ T cell counts as well as the cytotoxicity of T cells (Fan et al., 2019). These findings indicate that ExoPD-L1 contributes to immunosuppression by inducing T-cell dysfunction, suggesting that ExoPD-L1 might promote the disease progression of tumor patients (Theodoraki et al., 2018b; Fan et al., 2019).

Collectively, tumor ExoPD-L1 plays a pivotal part in mediating local and systemic immunosuppression in mouse models and tumor patients.

## Potential Clinical Implication of Circulating ExoPD-L1 as a Biomarker for Tumor Diagnosis, Disease Progression, and Immunotherapy Response

Tumor immunotherapy requires biomarkers for predicting disease progression, prognosis, clinical response, and the selection of suitable patients (Buder-Bakhaya and Hassel, 2018; Zhang et al., 2019). Tumor cell PD-L1 has been considered a predictor for response to immunotherapy in the clinic (Patel and Kurzrock, 2015; Aguiar et al., 2017; Lin et al., 2018; Li et al., 2019c). However, there are pitfalls of using cellular PD-L1 such as traumatic biopsy, missing small tumors, heterogeneity of PD-L1 expression within tumors, unavailability of dynamic observation, and limited sensitivity (Kaunitz et al., 2017; Bassanelli et al., 2018; Teixido et al., 2018; Davis and Patel, 2019; Stovgaard et al., 2019; Xu G. et al., 2019). Recent studies indicate that circulating ExoPD-L1 is emerging as a non-invasive and readily available biomarker, and is more easily detectable and reliable than both tissue and soluble PD-L1 in plasma (Liu et al., 2018b; Cordonnier et al., 2020; Huang et al., 2020; Pang et al., 2020).

### ExoPD-L1 as a Biomarker for Diagnosis and Disease Progression

Tumor-derived exosomes can be enriched from small volumes of patient plasma and are considered to be a potential biomarker based on liquid biopsy (Crow et al., 2019; Johnsen et al., 2019; Xie C. et al., 2019; Yekula et al., 2019, 2020; Brennan et al., 2020). The number and the protein content of exosomes in the blood of breast cancer patients are higher compared with those of healthy subjects and the increased exosome numbers positively correlates with tumor growth (Hesari et al., 2018). However, in melanoma, the level of ExoPD-L1 in the blood, rather than the number and total protein content of exosomes, is elevated in metastatic melanoma patients compared with healthy subjects (Chen G. et al., 2018). Furthermore, patients with NSCLC and adenocarcinoma also exhibit higher levels of circulating ExoPD-L1 compared with healthy controls (Liu et al., 2018b; Li et al., 2019a; Huang et al., 2020; Pang et al., 2020). Therefore, circulating ExoPD-L1 may be a potential diagnostic marker. In addition, high levels of circulating ExoPD-L1 were associated with metastatic melanoma, advanced HNSCC, and poor prognosis in pancreatic cancer, further indicating that circulating ExoPD-L1 may be a useful biomarker for tumor progression (Theodoraki et al., 2018a; Lux et al., 2019; Huang et al., 2020).

Soluble PD-L1 in plasma is also considered as a potential diagnostic and predictive biomarker for tumor recurrence and prognosis (Chatterjee et al., 2017; Okuma et al., 2017; Zhou et al., 2017; Chang et al., 2019; Shigemori et al., 2019; Liu S. et al., 2020). However, the levels of soluble PD-L1 were not different between NSCLC patients and healthy donors (Li et al., 2019a). Moreover, soluble PD-L1 did not correlate with disease progression in patients with metastatic gastric cancer, HNSCC, or NSCLC (Liu et al., 2018b; Fan et al., 2019; Li et al., 2019a; Pang et al., 2020). On the contrary, circulating ExoPD-L1 in plasma is an independent biomarker to predict poor prognosis in patients with metastatic gastric cancer (Fan et al., 2019). Furthermore, the levels of ExoPD-L1 correlated with the disease progression of patients with HNSCC and NSCLC, including tumor size, lymph node status, metastasis, and clinical stage (Theodoraki et al., 2018a, b; Li et al., 2019a).

Exosomal PD-L1 DNA is present in exosomes isolated from the plasma of glioblastoma patients (Ricklefs et al., 2018). The amount of ExoPD-L1 DNA from glioblastoma patients is associated with tumor volume, although the function of PD-L1 DNA remains unknown (Ricklefs et al., 2018; Lazaro-Ibanez et al., 2019). Together, liquid biopsy analysis of ExoPD-L1 protein and DNA in blood may provide biomarkers for tumor diagnosis and disease progression.

### ExoPD-L1 as a Biomarker for Efficacy of Anti-PD-1/PD-L1 Therapy

It is important to provide individualized precise treatment and to predict tumor response to immunotherapy (Madore et al., 2015; Kaunitz et al., 2017; Sui et al., 2018; Liu and Wu, 2019). To fulfill the need for real-time monitoring, the analysis of liquid biopsy-based circulating biomarkers is preferred (Nishino et al., 2013; Ando et al., 2019; Gregg et al., 2019; Kloten et al., 2019; Tang et al., 2020). Recent studies demonstrated that melanoma patients who were less responsive to anti-PD-1 blockade had a significantly higher level of circulating ExoPD-L1 prior to treatment as compared with responders (Chen G. et al., 2018). In addition, the increasing magnitude of circulating ExoPD-L1 in melanoma patients during early treatment periods can distinguish clinical responders from non-responders (Chen G. et al., 2018). Moreover, a prospective study on melanoma indicated that monitoring the levels of circulating ExoPD-L1 may be helpful to predict therapeutic efficacy and clinical outcome (Cordonnier et al., 2020). Additionally, compared with patients exhibiting recurrence, patients who did not relapse had higher levels of tumor-enriched CD3- ExoPD-L1 prior to therapy, which significantly decreased after five weeks of therapy (Theodoraki et al., 2018a, 2019). In contrast, tumor-enriched ExoPD-L1 levels increased at week five of therapy, whereas the CD3^+^ ExoPD-L1 levels decreased in patients with recurrence (Theodoraki et al., 2019). Thus, studying on the role of ExoPD-L1 derived from immune cells and tumor cells as biomarkers for tumor patients will be necessary. Interestingly, ExoPD-L1 mRNA can also be sequestered in exosomes of patient plasma in melanoma and NSCLC, and associated with response to anti-PD-1 inhibitors (Del Re et al., 2018; Zhao Z. et al., 2019).

Collectively, ExoPD-L1, including PD-L1 protein, DNA and mRNA, has the potential to become reliable biomarkers for immunotherapy. This is an effective complement to tumor PD-L1 and soluble PD-L1 to identify patients who may benefit from immunotherapy and to dynamically monitor therapeutic response (Chen G. et al., 2018; Theodoraki et al., 2019; Cordonnier et al., 2020; Daassi et al., 2020).

The regulation of PD-L1 expression is highly intricate and has been extensively addressed at transcriptional, posttranscriptional, translational, and posttranslational levels (Sun et al., 2018; Zerdes et al., 2018; Cha et al., 2019; Xu Y. et al., 2019; Fu et al., 2020; Han et al., 2020; Ju et al., 2020). Notably, soluble PD-L1 may be generated from ectodomain shedding mediated by either matrix metalloproteinases (MMPs) or a disintegrin and metalloproteases (ADAMs). Additionally, soluble PD-L1 may be produced by alternative splice variants omitting transmembrane domain (Dezutter-Dambuyant et al., 2016; Hira-Miyazawa et al., 2018; Aguirre et al., 2020; Orme et al., 2020; Romero et al., 2020). Although soluble PD-L1 is found in human serum and is regarded as a liquid biopsy predictor, whether soluble PD-L1 can deliver a regulatory signal through PD-1 remains elusive (Gu et al., 2018; Takeuchi et al., 2018; Abu Hejleh et al., 2019; Asanuma et al., 2020). Some studies reported that soluble PD-L1 inhibits T cell activation, while others suggested that soluble PD-L1 is likely to increase immune response by proteolytic reducing the amount of membrane-bound PD-L1 on both cell surface and exosome or by competing with membrane-bound PD-L1 for PD-1 binding (Dezutter-Dambuyant et al., 2016; Hira-Miyazawa et al., 2018; Aguirre et al., 2020; Romero et al., 2020). Furthermore, it is demonstrated that soluble PD-L1 produced by *CD274-L2A* splice variant lacks suppressive activity and functions as a PD-1 antagonist, suggesting the possibility that soluble PD-L1 might limit the immunoinhibitory effects of ExoPD-L1 (Wan et al., 2006; Steidl et al., 2011; Ng et al., 2019).

Additionally, PD-L2 is also expressed on tumor cells and involved in antitumor immune suppression (Latchman et al., 2001; Taube et al., 2014; Yearley et al., 2017; Larsen et al., 2019; Liao et al., 2019; Tanegashima et al., 2019; Nakayama et al., 2020). Moreover, PD-L2 not only possesses higher affinity for PD-1 than PD-L1 does but also may be highly coexpressed with PD-L1 in tumor cells and tissues (Youngnak et al., 2003; Cheng et al., 2013; Morales-Betanzos et al., 2017; Tang and Kim, 2019; Furuse et al., 2020; Wolkow et al., 2020). It is worth mentioning that proteolytic degradation or alternative splice variants also produce soluble PD-L2, which may be a complementary biomarker (He et al., 2004; Dai et al., 2014; Fukasawa et al., 2017; Costantini et al., 2018; Buderath et al., 2019; Wang Q. et al., 2019). Recently, a pilot study found PD-L2-expressing EVs in a murine sepsis model (Kawamoto et al., 2019). Moreover, sepsis patients displayed higher PD-L2 expression on EVs compared with healthy subjects (Kawamoto et al., 2019). Additionally, reduced exosomal PD-L2 was observed in IL-10-treated murine dendritic cells (Ruffner et al., 2009). However, it is unclear whether tumor cells are able to release exosomal PD-L2, and the function and regulation of exosomal PD-L2 in antitumor immunity are unexplored and worthy of further investigations (Solinas et al., 2020).

## Potential Methods for the Detection of Circulating ExoPD-L1 in Clinical Samples

A quick, simple, and sensitive assay is a prerequisite for a point-of-care test for ExoPD-L1 as a clinical biomarker. However, due to the small size and high heterogeneity of exosomes, the methods used most widely to detect ExoPD-L1 from tumor patients required ultracentrifugation and ELISA (Table 3). Therefore, low efficiency and sensitivity are two bottlenecks to the classic detection of ExoPD-L1 in the clinic (Liu et al., 2017; Yang et al., 2017). Efforts have been made to improve the sensitivity of the ELISA-based methods for detecting low levels of ExoPD-L1 (Liu et al., 2018a, b; Huang et al., 2020).

**TABLE 3:** Comparison of methods for detecting ExoPD-L1 in clinical samples.

Method	Instrument	Sample volume (μl)	Exosome isolation	Heterogeneous reaction system	Detection limitation	References
ELISA	Microplate reader	1000	Ultracentrifugation	Yes	200 pg/ml	Chen G. et al., 2018; Liu et al., 2018b; Theodoraki et al., 2018a, b, 2019; Fan et al., 2019; Li et al., 2019a; Lux et al., 2019; Cordonnier et al., 2020; Huang et al., 2020
HOLMES-Exo-PD-L1	Flow cytometer	1000	Ultracentrifugation	No	17.6 pg/ml	Huang et al., 2020
nPLEX assay	Compact SPR biosensor	50	Ultracentrifugation	No	Not given	Liu et al., 2018b
SERS immunoassay	Raman spectrometer	4	Fe_3_O_4_@TiO_2_ magnetic nanobeads	No	1 PD-L1^+^ exosome/μl	Pang et al., 2020

*ELISA, enzyme linked immunosorbent assay; HOLMES-ExoPD-L1, homogeneous, low-volume, efficient, and sensitive ExoPD-L1; nPLEX, nanoplasmonic exosome; SPR, surface plasmon resonance; SERS, surface-enhanced Raman scattering.*

### Ultracentrifugation-Based Methods

Recently, a homogeneous, low-volume, efficient, and sensitive ExoPD-L1 (HOLMES-ExoPD-L1) quantitation method has been developed (Huang et al., 2020). The HOLMES-ExoPD-L1 method combining PD-L1 aptamer with separation-free thermophoresis exhibits higher sensitivity and is more rapid than the classic ELISA-based methods (Lee et al., 2019; Huang et al., 2020). To completely surmount the disadvantages of ELISA, a nanoplasmonic exosome (nPLEX) assay has been established, which involves modified surface plasmon resonance (Enderle et al., 2015) with a compact SPR biosensor (Liu et al., 2018b). Notably, the nPLEX assay is able to detect ExoPD-L1 in 50 μl serum samples in real-time, which is undetectable by ELISA (Table 3; Liu et al., 2018b).

### Ultracentrifugation-Free Method

The methods described above are ultracentrifugation-based, time-consuming and yield low recovery (Momen-Heravi et al., 2013; Lobb et al., 2015). More recently, a quick and precise method for detecting ExoPD-L1 directly from clinical samples has been set up by coupling Fe_3_O_4_@TiO_2_ isolation with a surface-enhanced Raman scattering (SERS) immunoassay (Pang et al., 2020). Although it takes less than 40 min to complete the entire procedure, the separation efficiency for exosomes is 96.5% and the detection limit is one PD-L1^+^ exosome per microliter (Pang et al., 2020). Moreover, the number of ExoPD-L1 molecules in four μl of a patient’s serum is precisely quantified using this method (Pang et al., 2020). Overall, along with the advancement of novel technologies, there should have more methods in development. It should be noted that it is necessary to validate these methods in large cohorts before routinely using ExoPD-L1 as a clinical biomarker (Table 3).

## Potential Strategies Targeting ExoPD-L1 for Antitumor Therapy

Antibody blockade of PD-L1 is able to trigger an antitumor immune response, bringing about a persistent remission in a fraction of tumor patients. Recent studies have shown that the removal of ExoPD-L1 blocks tumor growth, even in mouse models which are resistant to anti-PD-L1 antibody (Chen G. et al., 2018; Yang et al., 2018; Kim et al., 2019; Poggio et al., 2019; Xie F. et al., 2019). This indicates that targeting exosome biogenesis inhibition and PD-L1 deletion represents an unexplored strategy for antitumor therapy.

### Blockade of Exosome Biogenesis Provides an Efficient Way to Overcome Resistance to Anti-PD-L1 Antibody

Immune checkpoint inhibitors are effective against various cancers, including melanoma, NSCLC, and renal cancer. However, the overall response rate in patients treated with anti-PD-1/PD-L1 antibodies is low (Page et al., 2014). In some tumor types, such as prostate cancer, the number of responders is very limited (Goswami et al., 2016; Sharma et al., 2017). However, genetic deletion of PD-L1 in TRAMP-C2 prostate cancer cells, which causes a reduction of both cell-surface PD-L1 and ExoPD-L1, strikingly prevents anti-PD-L1 antibody resistant tumor to grow in mice (Foster et al., 1997; Poggio et al., 2019). More importantly, the prostate cancer cells were also unable to grow in mice when Rab27a or aSNase2 was deleted by the CRISPR/Cas9 technique, leading to a blockade of exosome biogenesis (Poggio et al., 2019). Thus, targeting the process of exosome biogenesis may yield new approaches to overcoming tumor resistance to anti-PD-L1 antibodies.

**FIGURE 2:**
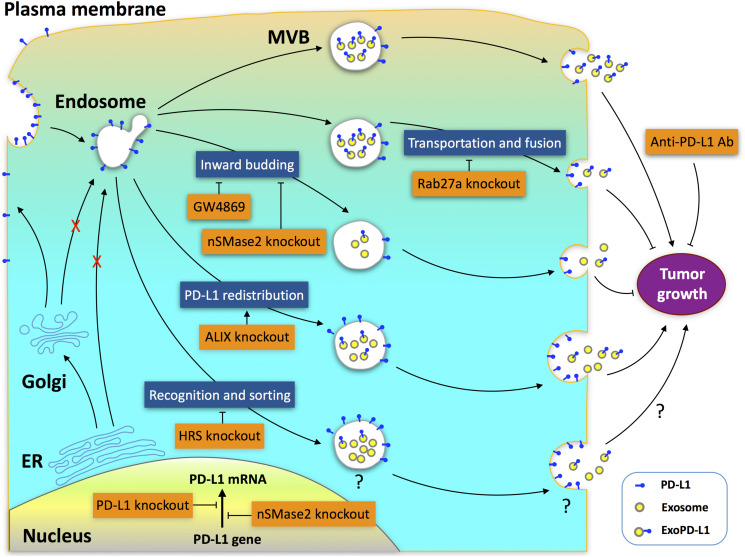
Potential targets for antitumor therapy in ExoPD-L1 biogenesis pathways. Multiple molecules, including Rab27a, nSMase2, ALIX, and HRS, participate in the complex processes of ExoPD-L1 biogenesis, which originates from the cell surface rather than from the ER or Golgi apparatus. Deletion of Rab27a decreases ExoPD-L1, but does not alter cell-surface PD-L1 levels. Deletion of nSMase2 reduces the levels of both cellular PD-L1 and ExoPD-L1 protein. Rab27a deletion causes a greater inhibition in exosome production compared with nSMase2 deletion, while nSMase2 deletion leads to a greater inhibition of ExoPD-L1 production compared with Rab27a deletion. GW4869, an inhibitor of nSMase2, inhibits ExoPD-L1 generation, but does not increase cellular PD-L1 levels. Knockdown of ALIX, which redistributes PD-L1 between the cell-surface and exosomes, results in a reduction of ExoPD-L1 production but an increase in cell-surface PD-L1. Blockade of Rab27a or nSMase2 results in suppression, whereas ALIX knockdown promotes tumor growth. Knockdown of HRS, an ESCRT-0 subunit, confers a decrease in ExoPD-L1 levels but an increase in cellular PD-L1 levels. The effects of HRS knockdown on the cell-surface PD-L1 levels and tumor growth remain unknown.

The biogenesis of ExoPD-L1 is a complicated process involving multiple molecules that impact the production of ExoPD-L1 in different ways (Chen G. et al., 2018; Monypenny et al., 2018; Yang et al., 2018; Poggio et al., 2019). The endosomal sorting complex required for transport complex (ESCRT) is a key mediator of MVB biogenesis (Henne et al., 2011; Matusek et al., 2014; Olmos and Carlton, 2016; Schoneberg et al., 2017; Furthauer, 2018). Hepatocyte growth factor-regulated tyrosine kinase substrate (HRS) is a subunit of ESCRT that mediates the recognition and sorting of exosomal cargos (Schmidt and Teis, 2012). Genetic deletion of HRS leads to a decrease in ExoPD-L1 levels but an increase in cellular PD-L1 levels. The effect of HRS blockade on tumor growth remains unknown (Chen G. et al., 2018). Apoptosis-linked gene 2-interacting protein X (ALIX), an ESCRT accessory protein, is a critical regulator potentially involved in the redistribution of PD-L1 between exosomes and cell-surface membranes (Baietti et al., 2012; Hurley and Odorizzi, 2012; Bissig and Gruenberg, 2014; Christ et al., 2017; Monypenny et al., 2018; Skowyra et al., 2018; Szymanska et al., 2018). Similar to nSMase2 or Rab27a deletion, ALIX knockdown also leads to a significant decrease in ExoPD-L1 production in breast cancer (Monypenny et al., 2018). However, in contrast to Rab27a or nSMase2 deletion, ALIX knockdown promotes, but does not suppress the tumor growth (Monypenny et al., 2018). The differential effects of these genetic deletions on cell-surface PD-L1 expression contribute to their different effects on tumor growth. Deletion of nSMase2 leads to a reduction in the levels of both cellular PD-L1 and ExoPD-L1 protein by downregulating the transcription of the PD-L1 gene (Poggio et al., 2019). Meanwhile, Rab27a deletion does not change cell-surface PD-L1 levels but causes a greater inhibition in exosome production compared with nSMase2 deletion (Poggio et al., 2019). Therefore, deletion of either nSMase2 or Rab27a completely inhibits tumor growth (Yang et al., 2018; Poggio et al., 2019). In contrast, ALIX knockdown leads to a significant increase in cell-surface PD-L1 on breast cancer cells, and thereby increases the aggressiveness of tumors (Monypenny et al., 2018). Collectively, the distribution of PD-L1 between exosomes and cell surfaces is pivotal for the efficacy of immunotherapy. Both ExoPD-L1 and cell-surface PD-L1 should be the focus of future therapeutic strategies (Figure 2).

### Combination of Exosome Biogenesis Inhibition With Anti-PD-L1 Antibody Enhances Immunotherapy Efficacy

It is a concern that the antitumor effect of removing ExoPD-L1 is not limited to the anti-PD-L1 resistant model of prostate cancer. Both Rab27a depletion and an nSMase2 inhibitor (GW4869) significantly inhibit the growth of breast cancer derived from 4T1 cells in mice, which is a drug-resistant model for breast cancer (Pulaski and Ostrand-Rosenberg, 2001; Lasso et al., 2019). These findings indicate that the blockade of exosome secretion is an effective tool to disrupt the growth of various tumors (Grasselly et al., 2018; Yang et al., 2018). Importantly, Rab27a knockdown and nSMase2 inhibition are more potent suppressors of breast cancer growth compared with anti-PD-L1 antibody treatment (Yang et al., 2018). More importantly, blocking either Rab27a or sMSase2 markedly enhances the therapeutic effectiveness of anti-PD-L1 antibody for the inhibition of breast cancer growth (Yang et al., 2018). Thus, combining exosome biogenesis inhibition with anti-PD-L1 antibody may be more potent for tumor suppression.

Rab27a knockout suppressed colorectal cancer growth and extended survival in MC38 mice, which is a colorectal cancer model exhibiting a partial response to anti-PD-L1 therapy (Deng et al., 2014; Poggio et al., 2019). In contrast to the resistant TRAMP-C2 prostate cancer model, either Rab27a knockout or anti-PD-L1 antibody blockade exhibited less of an effect on the MC38 colorectal cancer growth compared with PD-L1 genetic deletion (Poggio et al., 2019). However, the combination of exosome deletion with anti-PD-L1 antibody lengthened the lifespan of mice burdened with colorectal cancer to an extent similar to that of PD-L1 deletion (Poggio et al., 2019). Hence, ExoPD-L1 appears to impose an additional, but not redundant impact compared with anti-PD-L1 antibody on the suppression of tumor growth (Chen G. et al., 2018; Poggio et al., 2019). In addition, combined genetic deletion of Rab27a and PD-L1 showed a similar inhibition of tumor growth as compared with PD-L1 deletion, demonstrating that the inhibitory effect of exosomes on colorectal cancer growth occurs mainly through the deletion of ExoPD-L1 (Poggio et al., 2019). Thus, the combination of inhibitors targeting exosome secretion with anti-PD-L1 blockade targeting cell-surface PD-L1 may be a promising strategy to effectively suppress tumor growth in the clinic (Yang et al., 2018; Poggio et al., 2019).

### ExoPD-L1-Deficient Tumor Cells Induce Abscopal Effect and Antitumor Immune Memory

**FIGURE 3:**
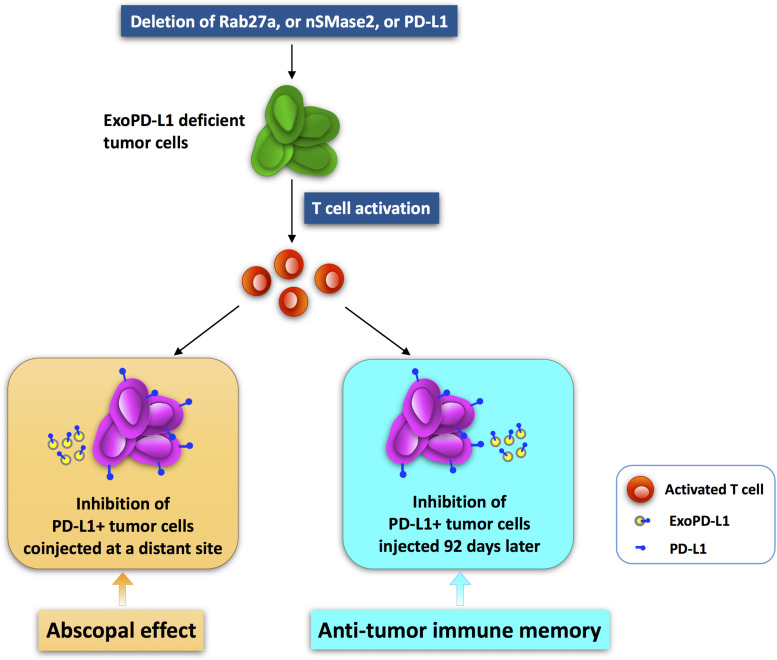
The abscopal effect and antitumor immune memory induced by ExoPD-L1-deficient tumor cells. Tumor cells with ExoPD-L1 deletion are generated by genetic mutation of Rab27a, nSMase2, or PD-L1. The growth of PD-L1-positive tumors at a distant site is inhibited when ExoPD-L1-deleted tumor cells are coinjected simultaneously. In addition, the growth of PD-L1-positive tumors injected secondarily 92 days later is also suppressed.

The abscopal effect refers to that treatment of a local tumor leads to the regression of distant tumors (Postow et al., 2012; Demaria and Formenti, 2020; Fionda et al., 2020; Mondini et al., 2020). This represents a promising therapeutic strategy for tumors and has drawn increased attention (Liu et al., 2018; Ngwa et al., 2018; Rodriguez-Ruiz et al., 2018; Choi et al., 2020). Interestingly, blocking ExoPD-L1 suppresses the growth of not only the local tumor, but also tumors at a distant site (Poggio et al., 2019). In the TRAMP-C2 mouse prostate cancer model, mutant cancer cells devoid of Rab27a, nSMase2, or PD-L1 expression completely failed to grow (Chen G. et al., 2018; Yang et al., 2018; Poggio et al., 2019). Surprisingly, the growth of WT tumor cells was reduced dramatically when the above mutant cells were injected simultaneously in the opposite sides of mice (Poggio et al., 2019). This indicates that ExoPD-L1-deficient tumor cells induce an abscopal effect on tumor growth (Poggio et al., 2019). On the contrary, WT cells had little or no effect on the growth of the mutant cells (Poggio et al., 2019). Furthermore, the numbers and activities of TILs in WT tumors in mice coinjected with ExoPD-L1-deleted mutant cells were significantly increased in comparison with the mice injected with WT cells alone (Poggio et al., 2019). Thereby, local anti-ExoPD-L1 treatment is able to induce a durable immune response to suppress the growth of tumors at distant sites (Figure 3).

Additionally, in the prostate cancer model, the mice injected with mutant cells deleted of PD-L1, Rab27a, or nSMase2 survived more than 90 days, whereas the mice injected with WT cells died soon or had to be euthanized because of tumors greater than 2 cm in diameter (Poggio et al., 2019). At 92 days after primary injection with mutant cells, surviving mice were reinjected with WT cells on the opposite flank. Interestingly, the WT prostate cancer cells were unable to grow in the mice that were preinjected with mutant cells (Poggio et al., 2019). In contrast, they grew normally in mice that were not preinjected with mutant cells (Poggio et al., 2019). Therefore, local import of tumor cells lacking of ExoPD-L1 induced a strong antitumor memory response against secondarily challenged tumor cells that secrete ExoPD-L1 (Poggio et al., 2019). In summary, the anti-ExoPD-L1 therapy that targets the tumor at one site may be of clinical significance due to the triggering of a systemic and durable immune response against tumors at multiple sites or challenged secondarily (Figure 3).

Currently, a dozen of small molecules have been recognized as exosome inhibitors (Luberto et al., 2002; Johnson et al., 2016; Catalano and O’Driscoll, 2020). GW4869 and Nexinhib-20 are inhibitors of nSMase2 and Rab27a, respectively (Luberto et al., 2002; Johnson et al., 2016). Moreover, Yang et al. reported that GW4869 inhibited exosome secretion of MDA-MB-231 human breast tumor cells *in vitro* and 4T1 mouse mammary tumor cells *in vitro* and *in vivo* (Yang et al., 2018). However, neither GW4869 nor Nexinhib-20 are able to inhibit exosome release in other tumor cell lines (Phuyal et al., 2014), although genetic deletion of either nSMase2 or Rab27a leads to tremendous loss of exosome secretion (Poggio et al., 2019). It has been established that exosomes participate in a variety of physiological processes and can be released from a variety of cell types (Colombo et al., 2014; Lo Cicero et al., 2015; van Niel et al., 2018; Catalano and O’Driscoll, 2020). It is likely that exosome inhibitors might interfere with normal cell functions by affecting the exosome release from healthy cells (Kalluri, 2016; Hessvik and Llorente, 2018; van Niel et al., 2018; Catalano and O’Driscoll, 2020; Hassanpour et al., 2020). Thus, it should be noted that exploitation of exosome inhibitors for tumor immunotherapy should be conducted with caution due to the potential adverse effects on healthy tissues (Dinkins et al., 2014). In addition, similar to immune checkpoint inhibitors, the restoration of T cell activation mediated by ExoPD-L1 blockade is non-specific and may result in immune-related adverse events (Cuzzubbo et al., 2017; Wanchoo et al., 2017; Barroso-Sousa et al., 2018; Myers, 2018; Sandigursky and Mor, 2018; Sibaud, 2018; Varricchi et al., 2018; Fan et al., 2020; Gauci et al., 2020; Liu T. et al., 2020; Ueki et al., 2020; Williams et al., 2020). Collectively, exosome inhibitors that selectively target cancer cells need to be developed to maximize the antitumor immune responses and minimize the possible side effects of blocking ExoPD-L1 release (Nagai and Muto, 2018; Weinmann and Pisetsky, 2019).

## Concluding Remarks

Exosomal PD-L1 derived from tumors is able to suppress antitumor immunity locally and systemically through ligation of PD-1 on T cells, which facilitates immune escape and tumor progression. Additionally, circulating ExoPD-L1 is emerging as a liquid biopsy biomarker for diagnosis, prognosis, stratifying eligible patients, and real-time monitoring of clinical response. Furthermore, the therapeutic strategies targeting ExoPD-L1 are potentially promising by inhibiting the biogenesis of PD-L1-expressing exosomes, and inducing the abscopal effect and antitumor memory response.

However, many issues remain to be resolved. First, understanding the mechanism by which cytokines regulate the biogenesis of ExoPD-L1 is needed. It has been reported that IFN-γ enhances ExoPD-L1 secretion by multiple tumors (Chen G. et al., 2018; Mimura et al., 2018; Monypenny et al., 2018; Poggio et al., 2019). Moreover, there is crosstalk between IFN-γ and epidermal growth factor in the regulation of the distribution of ExoPD-L1 and cellular PD-L1 in breast cancer cells (Monypenny et al., 2018). Recent studies have revealed that both cell-surface PD-L1 and ExoPD-L1 play crucial roles in immunosuppression, tumor progression, and response to cancer immunotherapy (Zou et al., 2016; Chen G. et al., 2018; Theodoraki et al., 2018b; Fan et al., 2019; Kim et al., 2019; Xie F. et al., 2019; Cordonnier et al., 2020; Huang et al., 2020; Tang et al., 2020). Nevertheless, the manner in which inflammatory cytokines affect PD-L1 expression on tumor cells and exosomes is still elusive (Akbay et al., 2013; Chen et al., 2015, 2019; Wang X. et al., 2017; Li et al., 2018; Lin et al., 2020). Second, studies regarding the origin of the PD-L1 nucleic acids in exosomes and their function in antitumor immunity are required. PD-L1 mRNA and DNA are found in exosomes derived from tumor cells in addition to PD-L1 protein (Del Re et al., 2018; Lubin et al., 2018; Ricklefs et al., 2018). It has been reported that RNA in cancer-derived exosomes is also relevant to the local and systemic interaction of exosomes with target cells (Skog et al., 2008; Matei et al., 2017; Yang et al., 2020). However, little is known about the role that ExoPD-L1 nucleic acids play, which should be addressed in the future. Third, immune cells and other cells in the tumor microenvironment or outside of the tumor also express PD-L1 and release exosomes. Moreover, the PD-L1 expression on immune cells is differently regulated and has an important impact on anticancer immunity (Kowanetz et al., 2018). However, the dynamics of host cell-derived ExoPD-L1 production and its potential function in immunosuppression remain unclear (Chen S. et al., 2018; Choo et al., 2018; Zhou et al., 2018; Carreras-Planella et al., 2019; Hong et al., 2019; Lan et al., 2019; Wang X. et al., 2019). Recent studies of murine tumor models indicate that PD-L1 expressed on host cells rather than on tumor cells is the primary target for immunotherapy, and determines the efficacy of the PD-1/PD-L1 blockade (Lin et al., 2018; Tang et al., 2018). Thereby, the immunoregulatory impact of ExoPD-L1 produced by host cells, such as T and B cells, macrophages, dendritic cells, epithelial cells, and mesenchymal stem cells, should be addressed. Finally, determining the regulatory role of ExoPD-L1 on various PD-1-expressing immune cells is of significant interest. In addition to T cells, PD-1 is also expressed on natural killer cells, macrophages, and dendritic cells, which are enriched in the tumor microenvironment (Karyampudi et al., 2016; Gordon et al., 2017; Hsu et al., 2018; Mariotti et al., 2019). Hence, the effect mediated by ExoPD-L1 on a group of PD-1-positive immune cells is a highly relevant issue in tumor immunology. We believe that a better understanding of the regulatory roles of ExoPD-L1 in host resistance to immunotherapies will offer novel therapeutic strategies for cancer patients in the future (Shergold et al., 2019).

## Author Contributions

KZ and FL were major contributors in searching the literature and writing the manuscript. SG and GL reviewed the manuscript and provided significant revisions. QS gave guidance for figures. All authors read and approved the final manuscript.

## Conflict of Interest

The authors declare that the research was conducted in the absence of any commercial or financial relationships that could be construed as a potential conflict of interest.
